# Hydrated Nucleus Pulposus Extrusion in Dogs: A Narrative Review of Pathophysiology, Diagnosis, and Clinical Management

**DOI:** 10.3390/ani16213516

**Published:** 2026-11-06

**Authors:** Alexandru-Gabriel Neagu, Ruxandra-Georgiana Pavel, Cătălin Micșa, Cristina Fernoagă, Niculae Tudor

**Affiliations:** Faculty of Veterinary Medicine, University of Agronomic Sciences and Veterinary Medicine of Bucharest, 105 Splaiul Independenței, 050097 Bucharest, Romania; alexandru.neagu@fmvb.usamv.ro (A.-G.N.); cristina.fernoaga@fmvb.usamv.ro (C.F.); niculae.tudor@fmvb.usamv.ro (N.T.)

**Keywords:** hydrated nucleus pulposus extrusion, intervertebral disc disease, magnetic resonance imaging, spinal cord compression, conservative management, dog

## Abstract

Hydrated nucleus pulposus extrusion (HNPE) is a non-Hansen type of acute canine intervertebral disc herniation: gelatinous, minimally degenerate nuclear material extrudes and forms a well-marginated extradural mass on MRI, the so-called ‘seagull sign’. It differs from classic Hansen type I or II disc disease, typically affecting the cervical spine of small-breed dogs, with spinal hyperaesthesia often mild or absent. This narrative review synthesises the literature on HNPE in dogs, focusing on four clinically relevant comparisons: cervical versus thoracolumbar disease, MRI versus computed tomography for diagnosis, conservative versus surgical management, and the proposed causes of extrusion from a non-degenerate disc. Across the studies reviewed, most dogs recover functionally whether managed surgically or conservatively, and recovery time relates more closely to intramedullary T2-weighted hyperintensity than to treatment choice. Gaps remain: comparative studies are retrospective and non-randomised, so treatment equivalence remains unproven, thoracolumbar cases remain fewer, and the terminology separating HNPE from acute non-compressive nucleus pulposus extrusion (ANNPE) is used inconsistently. We conclude that conservative management is a reasonable first option for ambulatory dogs with compressive HNPE and normal to mild hyperaesthesia, and that surgical decompression remains appropriate for severe or progressive deficits, and we set out priorities for future research.

## Simple Summary

Hydrated nucleus pulposus extrusion, or HNPE, causes sudden spinal cord compression in dogs and has only been clearly recognised as a condition in the last 10 years. It is not the classic extrusion most owners and veterinarians imagine when they hear intervertebral disc disease. In HNPE, soft, water-rich material escapes from a disc that is otherwise largely healthy and pushes suddenly against the spinal cord, usually in a small dog. The weakness or paralysis comes on within minutes, and surprisingly often with little or no pain. Because it behaves so differently from ordinary disc herniation, telling the two apart on examination and MRI changes what a veterinarian recommends and how an owner is counselled about surgery versus other treatments. This review brings together what is currently known about the causes, imaging appearance, treatment options and likely outcome of HNPE in dogs.

## 1. Introduction

Intervertebral disc disease (IVDD) is one of the most common neurological problems in dogs, in both first-opinion and referral practice. For decades, the condition was described almost entirely through Hansen’s original pathological scheme: type I extrusion, tied to chondroid metaplasia and seen mainly in chondrodystrophic breeds, and type II protrusion, tied to fibroid degeneration and seen mainly in older, non-chondrodystrophic dogs [1,2]. That binary picture does not fit every dog presenting with acute paresis after a disc-related event. In the last fifteen years, two further, biologically distinct entities have been carved out of what used to be lumped together as ‘atypical’ disc disease. Acute non-compressive nucleus pulposus extrusion (ANNPE) is a traumatic, high-velocity injury caused by largely non-degenerate material that does not persist as a compressive lesion. Hydrated nucleus pulposus extrusion (HNPE) involves similarly non-degenerate, water-rich material, but here it remains as a persistent, well-defined compressive mass [2,3].

Beltran et al. were first to characterise cervical HNPE in detail, describing a cohort of dogs with acute compressive cervical myelopathy whose magnetic resonance imaging (MRI) appearance did not match the usual Hansen-type pattern [1]. Recognition has grown steadily since then, in both the cervical and thoracolumbar spine. A run of retrospective series, comparative treatment studies and case reports, several published only in the last two or three years, has started to fill in its epidemiology, imaging features and response to treatment [4,5,6,7,8,9]. HNPE is still easy to miss in general practice and gets confused readily with classic disc extrusion or with ANNPE. As far as we are aware, it has not previously been the subject of a dedicated narrative synthesis.

The extruded material in HNPE is soft rather than firmly compacted, and the disc it came from is usually not markedly degenerate, which means that the condition tends to behave differently from classic disc extrusion. Several of the studies reviewed here report high recovery rates whether dogs are managed surgically or conservatively [4,5]. That changes how a case is discussed with an owner, what it costs, and how urgently referral for surgery gets pursued, especially in practices without advanced imaging close at hand. This review sets out to summarise the pathophysiology and proposed causes of HNPE in dogs, compare its cervical and thoracolumbar presentations, weigh MRI against computed tomography (CT) for diagnosis, compare the published evidence on conservative versus surgical management, and point out where the evidence base is still thin.

## 2. Materials and Methods

This is a narrative review rather than a systematic one, reflecting how young and scattered the literature on HNPE still is. We searched PubMed, Google Scholar and Web of Science using the terms ‘hydrated nucleus pulposus extrusion’, ‘hydrated nucleus pulposus herniation’, ‘acute non-compressive nucleus pulposus extrusion’ and ‘canine intervertebral disc disease’, each combined with ‘dog’, and hand-searched the reference lists of everything retrieved. HNPE cannot really be understood in isolation from the broader biology and management of canine disc disease, so the search was extended to disc degeneration biology and the genetics of chondrodystrophy, comparative diagnostic imaging, and surgical, conservative and rehabilitation outcomes for acute canine myelopathy. We prioritised material published between 2016 and 2026, weighted toward the last 6–7 years, since that is where most of the clinically useful, larger studies have been published. A handful of earlier sources were kept as deliberate exceptions where nothing more recent covers the same ground: the 2012 paper that first characterised HNPE, a 2015 prospective study offering the only detailed MRI–surgery correlation currently available, a 2015 review of peracute non-compressive disc extrusions, and foundational 2013 histological work on canine disc degeneration. We included peer-reviewed original research, retrospective and prospective case series, comparative treatment studies, consensus statements and case reports that provided sufficiently detailed clinical information, plus a small number of directly comparative feline reports retained for context. Conference abstracts without accompanying data, errata and editorials, and papers not substantively about HNPE, ANNPE, their differential diagnosis or immediate clinical context were excluded. No formal risk-of-bias scoring or meta-analysis was attempted, in keeping with the narrative design, but it should be said plainly that the level of evidence here is almost entirely limited to retrospective case series.

The searches were run between July and August 2026. In PubMed, limited to records indexed up to 31 July 2026, the four terms combined with ‘dog’ returned 22, 19, 14 and 9 records, respectively (64 in total), and 43 unique records remained after duplicates were removed. Their titles and abstracts were screened against the criteria above by three of the authors, and 26 were excluded: 17 did not deal with HNPE, ANNPE or their differential diagnosis, 5 were older studies superseded by more recent work, 3 were limited to ANNPE or fibrocartilaginous embolic myelopathy and were already covered by other sources, and 1 was an editorial. The remaining 17 records were included. In Google Scholar, searching titles only and limited to records published up to 31 July 2026, the four terms returned 26, 1, 19 and 29 records (75 in total). In Web of Science, with the same limit, the four terms combined with ‘dog’ returned 28, 18, 26 and 181 records (253 in total). The Google Scholar and Web of Science results were also checked by the same three authors, who added further relevant publications not retrieved by PubMed, mainly on the background topics listed above. Together with the reference lists of the papers already retrieved, these sources provided 36 references. In total, 53 references were included: 17 from the PubMed screening and 36 from Google Scholar, Web of Science and reference lists.

## 3. Pathophysiology and Etiopathogenesis

### 3.1. Mechanism of Extrusion

The mechanism behind HNPE looks quite different from classic Hansen type I or type II disease. In chondrodystrophic dogs, chondroid metaplasia of the nucleus pulposus drives progressive dehydration, mineralisation and loss of elasticity. When the disc fails, what extrudes through the degenerate annulus is firm and often mineralised [1,2]. HNPE material, by contrast, is gelatinous, translucent and almost water-like. In the prospective series of Dolera et al., cytology of material collected at surgery showed poorly cellular, amorphous basophilic material with only occasional neutrophils and no infectious agents or atypical cells; the material was too liquid for histology [10]. These findings describe the extruded material, not the disc it came from. They make an infectious or neoplastic process unlikely, but on their own they cannot show that the disc was non-degenerate. As far as we know, the disc of origin has not been examined histologically in any HNPE series, and the case for a non- or minimally degenerate disc rests mainly on MRI, where the affected disc usually keeps a normal or near-normal signal and height [1,10]. Even this is not always so: one comparative study found degenerative changes in the disc of origin in 60% of dogs with thoracolumbar HNPE [7], and the histology of material removed in a single thoracolumbar case is discussed in Section 5.2. Dolera et al. also compared MRI with surgical findings in 36 dogs with cervical HNPE and found that the extruded material typically sits beneath the dorsal longitudinal ligament, which is lifted dorsally but stays intact over the compression. That may explain both the sharply demarcated appearance on MRI and why the lesion tends to stay put rather than migrate widely within the canal [10].

### 3.2. Proposed Causes and Contributing Factors

What actually triggers extrusion of material that has not undergone the usual degenerative changes remains one of the least settled questions in HNPE. One plausible explanation is a brief, sharp rise in intradiscal pressure during an otherwise ordinary movement, such as a jump, an awkward twist or a sudden turn of the neck, acting on a disc with a small, inconspicuous annular defect and pushing nuclear material out before chondroid degeneration has had time to develop [1,4]. This fits how owners typically describe the onset: sudden, often during nothing more dramatic than normal play. Because the material is soft rather than firm, it has been suggested that it disperses or is resorbed within the vertebral canal more readily than mineralised Hansen type I material [4,5]. If so, this would be a biologically plausible reason why conservative management does comparatively well in several of the series discussed below. For now, though, resorption remains largely a hypothesis. Neither Borlace et al. nor Nessler et al. performed systematic follow-up MRI [4,5], so improvement in a conservatively managed dog does not tell us what happened to the material. The only direct imaging evidence in these cohorts is a single conservatively managed dog, euthanised four days after presentation, in which the extruded material could no longer be seen on post-mortem MRI [5].

A second, complementary explanation looks at anatomy rather than disc biology. The cervical spine allows far more motion than the thoracolumbar spine, where rib and costovertebral attachments limit flexion, extension and rotation at each segment. This increased mobility, particularly pronounced in small dogs at levels such as C2–C3 and C3–C4, may result in greater mechanical stress even in the absence of pre-existing degeneration, potentially explaining why cervical HNPE outnumbers thoracolumbar HNPE in most published series [5], although thoracolumbar cases predominated in one referral population [6]. There also seems to be a breed effect alongside the biomechanical one: HNPE keeps turning up in small and toy breeds that are not classically chondrodystrophic. That argues against chondroid metaplasia as a shared cause and points instead toward some other, still uncharacterised predisposition such as connective tissue laxity, disc composition, or some structural weak point in these breeds not yet identified [1,8]. Part of the difficulty in pinning down a single cause may simply be conceptual rather than biological. HNPE, ANNPE and, at the milder end, some non-compressive presentations have been proposed to sit along one continuum of acute nuclear extrusion, varying in how much material is released and whether it stays compressive, rather than representing entirely separate disease processes. The terminology for this continuum is still not applied consistently across the literature [2,11].

### 3.3. Disc Biology and Genetic Background

To understand why HNPE-affected discs behave so differently from classically degenerate ones, it helps to step back to normal disc biology first. The healthy canine nucleus pulposus is populated by notochordal cells that keep its water content and gel-like consistency. Disc degeneration is defined histologically by the gradual replacement of these cells with chondrocyte-like cells, alongside progressive dehydration and loss of proteoglycan content [12]. Chondrodystrophic breeds lose their notochordal cell population early in life and undergo widespread chondroid metaplasia across most of their discs by young adulthood. Non-chondrodystrophic breeds typically retain notochordal cells for much longer and degenerate later and more focally. A complementary histological study demonstrated that fibroid degeneration in non-chondrodystrophic dogs does not represent a distinct pathological process from chondroid degeneration, but rather appears to reflect a milder or more advanced stage of the same underlying degenerative process [13,14]. There is now a specific genetic mechanism behind much of this breed difference: retrogene insertions near the FGF4 locus drive both the chondrodystrophic phenotype and a substantially earlier age of onset and higher lifetime risk of intervertebral disc herniation, in a dose-dependent manner across breeds [15]. Genotype-phenotype evidence from young dogs of a single breed makes the point sharply: FGF4-retrogene-positive individuals already show near-complete notochordal cell loss and cartilaginous replacement of the nucleus pulposus at an age when genotype-negative dogs still have an essentially normal, notochordal disc architecture [16]. Against this backdrop, HNPE looks less like a variant of classic degenerative disease, and more like a mechanically driven failure of an otherwise notochordal or near-normal disc. That fits modern classification schemes, which treat HNPE and ANNPE as biologically distinct from Hansen type I and type II disease rather than unusual presentations of the same process [17].

The link between HNPE and FGF4 status is, however, an inference. To our knowledge, no study has genotyped dogs with HNPE for the FGF4 retrogene insertions, so the connection rests on breed-level phenotype and on imaging and histological data from other dogs, not on genetic evidence from HNPE cases themselves. The phenotypic data do not point to a simple relationship either. Chondrodystrophic breeds made up 13 of 36 dogs with HNPE in one series and were less common among cervical than thoracolumbar cases [6]; in another study, 62% of dogs with thoracolumbar HNPE belonged to chondrodystrophic breeds, compared with 91% of dogs with thoracolumbar Hansen type I extrusion [7]. Breed is also a poor stand-in for genotype, since the FGF4 retrogene insertions segregate within many breeds [15]. Genotyping HNPE cases against breed-matched controls would be a simple way to find out whether FGF4 status affects the risk, or the site, of HNPE.

## 4. Epidemiology and Signalment

Cervical HNPE turns up most consistently in small and toy-breed dogs, including several breeds not otherwise associated with chondrodystrophic disc disease, though occasional chondrodystrophic breeds appear in the published series as well [1,8,10]. Most affected dogs are middle-aged to older, but the reported age range is wide. In the comparative series by Nessler et al., 33 of 36 dogs with HNPE had a cervical lesion, and only three had a thoracolumbar lesion [5], although the balance differs between centres, and thoracolumbar cases outnumbered cervical ones (21 versus 15 dogs) in another referral series [6]. More recent work directly comparing thoracolumbar HNPE with both the cervical form and classic thoracolumbar disc extrusion suggests the underlying imaging and pathological picture does not differ greatly between sites, even though signalment and clinical severity can differ [6,7]. No consistent sex predisposition has emerged from the literature. Age alone should not be relied on to exclude HNPE, either: it has now been documented in a 4-month-old cross-breed dog with T13-L1 spinal cord compression, considerably younger than the signalment usually described [18]. That keeps HNPE on the differential list for acute paresis in young dogs where classic degenerative disc disease would otherwise seem unlikely, even allowing for the fact that a disc this young is still physiologically hydrated and notochordal on its own. For context, classic IVDD as a whole is common across the general dog population. A large owner-reported survey involving more than 43,000 companion dogs in the United States estimated the overall lifetime prevalence to be in the low single digits, while identifying several demographic and lifestyle-associated risk factors. Breed-specific studies further indicate substantial variation in both prevalence and risk profiles within individual breeds. For example, a recent analysis of English Cocker Spaniels identified breed-specific patterns of disease occurrence and associated risk factors [19,20]. HNPE almost certainly accounts for only a small slice of this overall disease, but exactly how it compares with classic Hansen type I and II disease has not been formally established in any published series.

### Regional Distribution: Cervical Versus Thoracolumbar HNPE

Because most published cases are cervical, the thoracolumbar form has been described in fewer studies, although two comparative series have now characterised it in more detail [6,7]. Still, the pattern across the comparative studies is one of broad similarity rather than a fundamentally different disease. Imaging appearance and apparent responsiveness to both conservative and surgical management look much the same regardless of location. The two forms differ mainly in frequency and, to a lesser extent, in signalment and pain: in one series, thoracolumbar HNPE occurred more often in chondrodystrophic breeds and was more often painful and lateralised than cervical HNPE [6]. Table 1 summarises the principal points of comparison between cervical and thoracolumbar HNPE as currently reported.

## 5. Clinical Presentation

### 5.1. Cervical HNPE

Dogs with cervical HNPE typically present with peracute tetraparesis or tetraplegia, sometimes preceded by a brief vocalisation, but often without ongoing spinal hyperaesthesia by the time they are examined. In the prospective series of Dolera et al., three-quarters of the dogs showed no pain at presentation, and the rest were only mildly painful [10]. Compared with dogs with other cervical myelopathies, dogs with HNPE were more often non-ambulatory (89% versus 54%), and they were less often painful than dogs with cervical Hansen type I extrusion [8]. Deficits range from mild paresis to non-ambulatory tetraplegia. As far as we know, no study has formally tested whether the degree of cord compression relates to the severity of deficits at presentation; the studies that measured compression looked at its relationship with recovery instead (Section 6.1) [5]. Problems with voluntary urination affect a minority of dogs: urinary retention was recorded in 5 of 30 dogs with available data in one series [5], and 4 of 16 surgically treated dogs could not urinate voluntarily in another [4]. Respiratory compromise, sometimes requiring mechanical ventilation, has been described in the most severely affected dogs [8,10]. Horner’s syndrome is not specific to cranial cervical lesions: because the descending sympathetic pathways run through the lateral funiculi along the whole cervical cord, it can occur with severe lesions at any cervical level, as well as with lesions of the T1–T3 segments, but we did not find it reported in the HNPE series reviewed here. The clinical picture can be broader still: one case report described dysphonia as the presenting sign in a dog with C4–C5 HNPE and suspected progressive myelomalacia, a reminder that cervical HNPE can occasionally look like a laryngeal or upper airway problem before its neurological origin becomes clear [21].

### 5.2. Thoracolumbar HNPE

Thoracolumbar HNPE is reported far less often than the cervical form, but the clinical picture follows the same basic pattern: peracute onset of paraparesis or paraplegia, usually with little or no spinal pain, in a dog whose disc does not show the marked degeneration typical of classic thoracolumbar Hansen type I extrusion [6,7]. Alcoverro et al. found that thoracolumbar HNPE can be told apart from classic thoracolumbar disc extrusion using both the clinical picture (pain score and breed included) and the MRI signal characteristics of the lesion [7]. However, the limited number of published thoracolumbar cases means that these distinctions should be interpreted cautiously and considered preliminary.

Beyond the larger series summarised above, individual case reports have done a good deal to broaden the recognised clinical spectrum of HNPE. There is a thoracolumbar (L2–L3) case treated by hemilaminectomy, in which the retrieved material was partly firm and histologically consistent with a partially degenerated nucleus pulposus that still contained notochordal cells [22], an unusual double, adjacent-level cervical presentation where the imaging picture was considerably more complex than the single well-demarcated lesion typically described [23], and, from our own group, a juvenile thoracolumbar case in a 4-month-old cross-breed dog with T13-L1 spinal cord compression whose MRI features fit HNPE rather than classic Hansen-type extrusion [18]. None of these amounts to much on its own, but read alongside the larger series, they suggest HNPE can turn up across a wider range of ages, spinal locations and imaging patterns than any single study would indicate.

## 6. Diagnostic Imaging

### 6.1. Magnetic Resonance Imaging (MRI)

**Figure 1:**
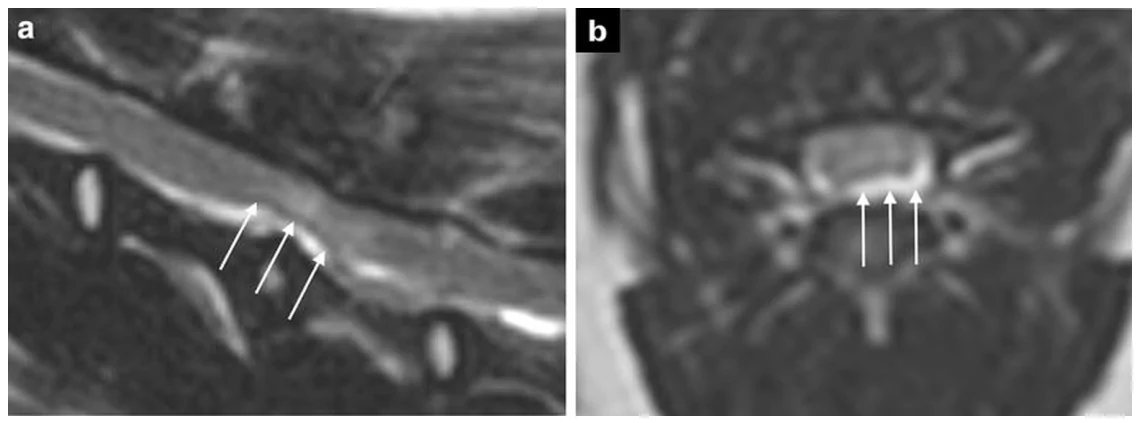
Magnetic resonance images of a dog with cervical HNPE at C4–C5 causing mild spinal cord compression. (**a**) Sagittal T2-weighted turbo spin-echo image showing the seagull sign; arrows mark the point where the extruded material lifts the dorsal longitudinal ligament from the floor of the vertebral canal, giving the typical double-winged outline. (**b**) Transverse T2-weighted turbo spin-echo image of the same dog at the level of the lesion; arrows mark the compressive extradural material, which in this plane appears as a focal, well-defined extradural mass without the gull-wing shape. Adapted from Dolera et al. [10], distributed under the terms of the Creative Commons Attribution 4.0 International License (http://creativecommons.org/licenses/by/4.0/).

MRI is the imaging method of choice for HNPE, and it is largely what lets the condition be distinguished from classic disc disease in the first place. The lesion typically appears as a well-marginated extradural mass, hyperintense on T2-weighted sequences and isointense to hypointense on T1-weighted sequences relative to the spinal cord, often spreading craniocaudally in a shape commonly likened to a seagull or a bird’s wing as it lies dorsal to the vertebral bodies [1,5,10] (Figure 1a). This appearance shows up specifically on sagittal images, where the craniocaudal extent of the lesion can be appreciated. On transverse sections, at any given level, the lesion instead looks like just a focal, well-marginated extradural mass usually ventral or ventrolateral, without the gull-wing shape that only becomes visible on the sagittal plane [1,10] (Figure 1b). The disc of origin itself often looks normal or only mildly degenerate on MRI, in contrast to the decreased T2-weighted signal and reduced intervertebral disc space commonly observed at sites of Hansen type I extrusion [1,10]. Spinal cord compression varies widely, from 13.5% to 57.2% of the cross-sectional area in a prospective series [10] and from 0% to about 62% in a retrospective one [5]. It matters which outcome each study looked at. Nessler et al. found no association between compression and either the time taken to walk again or short-term survival; the only MRI variable linked to recovery was the length of intramedullary T2-weighted hyperintensity in cervical cases, which was associated with regaining ambulation within nine days and with short-term survival [5]. So far, then, compression has been tested against recovery time and short-term survival rather than against the severity of deficits at presentation, and it is best treated as one imaging finding among several rather than as a predictor of outcome. A consensus review by the Canine Spinal Cord Injury Consortium discusses this imaging appearance in the broader context of diagnostic modality selection for intervertebral disc disease. Similarly, a related specialist consensus statement recommends cross-sectional imaging over conventional radiography when acute disc extrusion is suspected [24,25]. Sequence selection also matters for lesion characterisation. Three-dimensional T2-weighted fast spin-echo sequences perform at least as well as conventional two-dimensional sequences and may offer some advantages when assessing thoracolumbar disc lesions in small-breed dogs [26]. In a prospective study of 20 non-ambulatory dogs under 15 kg with suspected thoracolumbar intervertebral disc extrusion (not HNPE), the length over which the cerebrospinal fluid (CSF) signal was attenuated on the half-Fourier acquisition single-shot turbo spin-echo (HASTE) sequence, expressed relative to the length of the second lumbar vertebra, differed significantly between dogs with and without deep pain perception, but was not associated with neurological grade [27]. Quick, targeted sequences of this kind may therefore add prognostic information, although they have not yet been studied in dogs with HNPE.

Diagnostic accuracy also varies according to MRI field strength. Low-field MRI has demonstrated reasonable, although not consistently reliable, accuracy in differentiating disc extrusion from protrusion at the lumbosacral level, highlighting a potential limitation for practices without access to high-field MRI systems [28]. MRI is not entirely reliable, as its accuracy in identifying suspected progressive myelomalacia decreases when characteristic clinical signs are absent at the time of imaging. Therefore, imaging findings should always be interpreted in conjunction with the clinical presentation and other diagnostic findings, rather than considered in isolation [29]. MRI of the brain or spine in dogs and cats requires general anaesthesia, introducing additional management considerations, including careful thermal and cardiorespiratory monitoring. These concerns are particularly relevant in small-breed dogs, which account for the majority of reported HNPE cases.

### 6.2. Computed Tomography (CT) and Myelography

In addition to MRI, compressive HNPE has been identified using multislice computed tomography (CT) and CT myelography. Royaux et al. demonstrated that an extradural mass of soft-tissue attenuation causing spinal cord compression could be detected with these techniques, providing a valuable alternative when MRI is unavailable or contraindicated [30]. However, HNPE material may exhibit attenuation similar to that of the spinal cord on non-contrast CT, limiting lesion conspicuity and potentially necessitating conventional myelography or CT myelography for more reliable identification. In a review of myelo-CT findings in 15 surgically treated dogs, Farré Mariné et al. further highlighted the limitations of CT in fully characterising subtle lesions, for which MRI may provide superior diagnostic detail [31]. CT myelography may also help identify atypical extrusion patterns that are difficult to characterise using conventional imaging approaches. However, broader evaluations of the CT literature suggest that, despite its diagnostic value, CT remains less reliable than MRI for the detection and characterisation of intervertebral disc extrusion [32,33]. Given these limits, CT-based diagnosis probably deserves some caution in practices without MRI access. Referral for MRI is worth pursuing whenever it is feasible, especially in dogs with marked or worsening neurological signs.

CT and CT myelography mainly show that an extradural compressive lesion is present, where it is and how far it extends; they do not show what it is. A non-mineralised extradural mass of soft-tissue attenuation, or an extradural filling defect on myelography, can also be caused by non-mineralised Hansen type I extrusion, epidural haemorrhage, epidural empyema, neoplasia or cystic lesions, and on myelography it is not always clear whether a lesion is extradural, intradural or intramedullary [24,32]. Without MRI, a CT-based diagnosis of HNPE should therefore be considered presumptive. It relies on the overall picture, namely a peracute and often non-painful onset, a well-defined ventral extradural lesion centred over a disc space and no mineralised material in the canal, and can only be confirmed at surgery or on later MRI [24,30,31].

### 6.3. MRI Versus CT: Comparative Diagnostic Value

Set side by side, the two modalities are best thought of as complementary, not interchangeable. MRI enables direct visualisation of the seagull sign and identification of the disc of origin without the need for contrast administration. These features are particularly valuable for differentiating HNPE from Hansen type I and type II disc disease and ANNPE. Accordingly, MRI should be considered the preferred diagnostic modality whenever available [1,10]. CT and CT-myelography remain a legitimate alternative, particularly where MRI is unavailable, too costly for the owner, or contraindicated. But because HNPE material is isoattenuating on plain CT, a confident diagnosis often needs myelographic contrast to be added, and even then some subtler cases resist full characterisation [30,31]. Table 2 sets the two modalities against each other across the features most relevant to diagnosing HNPE in practice.

## 7. Differential Diagnosis

Telling HNPE apart from the other causes of acute myelopathy matters both for how a case is managed and for how it gets explained to an owner. The main differentials are classic Hansen type I extrusion, Hansen type II protrusion, and ANNPE, alongside fibrocartilaginous embolic myelopathy, discal cysts and, less often, neoplastic or inflammatory causes of acute spinal cord dysfunction [2,3]. Table 3 sets out the main distinguishing features of these entities as described in the reviewed literature.

HNPE and ANNPE are not always easy to separate cleanly, particularly when the compressive component is minimal. Several authors have suggested the two may sit on a continuum rather than being wholly distinct diagnoses, and the terminology across the literature has not always been applied consistently [2,3,34]. The overlap with intraspinal discal cysts is a particularly persistent source of confusion: a case series of 20 dogs with acute compressive cervical myelopathy argued that lesions previously labelled as discal cysts on imaging are, in most instances, better understood as HNPE, which matters for how older case reports using that terminology should now be read [35]. ANNPE is not confined to dogs, either. A series of 11 cats with presumptive ANNPE described a clinical picture and outcome much like that seen in dogs, suggesting the underlying mechanism is not species-specific, even though the great majority of published cases remain canine [36]. Concurrent or incidental spinal abnormalities have also been documented, including vertebral neoplasia in cases presenting with an ANNPE-like clinical and imaging pattern. Such findings highlight the importance of comprehensive diagnostic evaluation, even when imaging features appear characteristic of a specific condition [37].

Classic Hansen-type disease still needs to be actively ruled out on imaging rather than assumed absent. A retrospective series of 12 dogs with cervical intervertebral disc disease imaged by MRI found Hansen type I extrusions concentrated at C2-C3 and type II protrusions predominating at C6-C7, both distinguished from HNPE by more pronounced disc degeneration and a less sharply demarcated extradural lesion [38]. Developmental and structural causes of cervical pain or tetraparesis in small and toy breeds belong on the same differential list. Atlantoaxial instability, for example, is well recognised in Yorkshire Terriers and other toy breeds, distinguished from HNPE by its typically younger age at onset and by radiographic or CT evidence of dens or ligamentous abnormality rather than a soft-tissue extradural mass [39]. Spinal arachnoid diverticula should also be considered in the differential diagnosis, as they typically cause slowly progressive myelopathy rather than the peracute clinical presentation characteristic of HNPE. On MRI, they are distinguished by a cerebrospinal fluid-filled expansion of the subarachnoid space, rather than the extradural mass associated with HNPE [40]. Inflammatory central nervous system disease, such as meningoencephalitis of unknown origin, is a further differential worth bearing in mind, particularly in young or toy-breed dogs presenting with atypical or multifocal neurological signs [41].

## 8. Treatment

### 8.1. Conservative Management

Conservative management of HNPE typically involves strict activity restriction, analgesia as required, appropriate nursing care, including bladder management in non-ambulatory dogs, and a structured physiotherapy or rehabilitation programme [4,5]. These measures are consistent with specialist consensus recommendations for acute thoracolumbar intervertebral disc extrusion, which similarly emphasise activity restriction, pain control and supportive nursing care in appropriately selected patients [25]. Comparative studies discussed below indicate that dogs with compressive cervical HNPE managed conservatively may achieve functional outcomes comparable to those treated surgically, provided that case selection is appropriate. Conservative treatment is generally considered most suitable for ambulatory dogs or those with only mild ambulatory impairment and no evidence of progressive neurological deterioration [4,5]. Similar findings have been reported in small, non-brachycephalic dogs treated conservatively for acute thoracolumbar disc extrusion, with the majority regaining the ability to walk without surgical intervention [42].

Ambulatory status alone does not reflect how compressed the cord is, so it is worth spelling out how MRI findings influenced treatment choice in the comparative studies. In the two earliest studies, compression was not a formal selection criterion. Borlace et al. left the choice of treatment to the owners, and the median residual spinal cord area at the point of maximal compression was the same in the medically and surgically treated groups [4]. Nessler et al. likewise found no significant difference in compression between groups, although the MRI appearance of the extruded material seemed to influence the surgeon, with heterogeneous material more often treated surgically [5]. In another series, by contrast, treatment choice was significantly associated with the degree of compression [6]. MRI findings should therefore be considered together with ambulatory status and the course of the neurological signs, but no compression threshold has yet been validated to guide the choice between conservative and surgical treatment in HNPE.

A 2025 study by Phillips and Freeman provides a further perspective, reporting no apparent association between the strictness of exercise restriction during recovery and clinical outcomes in dogs diagnosed with ANNPE, fibrocartilaginous embolism or HNPE [9]. Although these findings challenge the assumption that stringent activity restriction is essential in every case, further studies are needed to confirm their applicability before current clinical recommendations can be reconsidered [9].

### 8.2. Surgical Decompression

Surgical decompression remains indicated in dogs presenting with severe, progressive or rapidly deteriorating neurological deficits, with the ventral slot approach typically used for cervical lesions and hemilaminectomy for thoracolumbar lesions. Surgery may also be favoured when rapid relief of spinal cord compression is desired, rather than relying on the slower response associated with conservative management [4,5]. At surgery, the extruded material is usually gelatinous or fluid and lies beneath the dorsal longitudinal ligament. Only one study described how the ligament was handled: it had to be opened in all 36 dogs to reach the material, through a small incision in 23 and by complete excision in 13 [10]. The other comparative studies did not report this [4,5]. Whether the ligament needs to be opened probably depends on where the material lies and whether it is lateralised, on the surgical approach (ventral slot or hemilaminectomy) and on what is found at surgery, so it should not be seen as a fixed requirement. Surgical technique and aftercare keep being refined. A single ventral slot has been shown to adequately address multiple concurrent cervical sites in the same dog. A large retrospective review of 593 cervical ventral slot cases helped quantify acute post-operative complication rates and the influence of prophylactic fenestration and chondrodystrophic status. Early postoperative hydrotherapy, started within days of thoracolumbar hemilaminectomy, does not appear to increase surgical site complications [43,44,45]. In dogs with suspected progressive myelomalacia, the optimal extent of surgical decompression remains a subject of debate. One comparative study reported similar outcomes following extensive hemilaminectomy and durotomy performed with either non-limited or cranially limited decompression [46]. Technique is not the only thing that determines surgical outcome. Older patients make up a meaningful share of dogs presenting with HNPE, and careful cardiovascular and respiratory monitoring throughout the perianaesthetic period remains an important part of getting a good result.

### 8.3. Comparative Outcomes

The most direct evidence comparing the two treatment approaches comes from two retrospective cohorts. Borlace et al. compared 18 conservatively managed dogs with 16 treated surgically for cervical HNPE, and found no significant difference in short- or long-term neurological outcome between the groups [4]. Of the 30 dogs with long-term follow-up available, all were neurologically normal, and only two of the conservatively managed dogs went on to need delayed surgery because they had not responded adequately to medical therapy. Nessler et al., working with a series of 36 dogs (33 cervical, 3 thoracolumbar), found much the same pattern, with functional recovery in 16 of 18 surgically managed dogs and 17 of 18 medically managed dogs [5]. Among the variables assessed, only the length of intramedullary T2-weighted hyperintensity in cervical cases was associated with regaining ambulation within nine days, and treatment, age, neurological status and degree of compression were not [5].

These results do not show that the two treatments are equally effective. A non-significant difference in small retrospective cohorts is not evidence of equivalence; Borlace et al. calculated that 174 dogs would have been needed to detect a difference in the rate of successful outcome, and neither study was designed to test equivalence or non-inferiority [4,5]. Treatment was not randomised but chosen by owners and clinicians, so selection bias cannot be ruled out: in one study the MRI appearance of the material influenced the decision to operate [5], and in another, cervical hyperaesthesia was more common in the surgically treated dogs [4]. Initial neurological severity did not differ significantly between groups, but the groups were small, very few dogs were tetraplegic or lacked nociception, and outcome definitions and follow-up times varied between studies [4,5]. Together with the findings of Phillips and Freeman on exercise restriction [9], the evidence suggests that outcome in HNPE depends largely on the lesion itself, particularly on intramedullary spinal cord changes, but it does not establish that conservative and surgical management are equivalent, especially in severely affected dogs. Table 4 brings these points together.

### 8.4. Practical Criteria for Case Selection

Put the evidence above into a short, practical checklist, and conservative management alone is most likely to succeed when the dog is ambulatory or only mildly non-ambulatory at presentation, neurological signs are static or improving rather than worsening, spinal hyperaesthesia is absent or mild, owners can commit to strict activity restriction and nursing care for several weeks, and timely re-assessment is available so a lack of response gets caught early rather than late. Surgical decompression is generally preferred in dogs with severe neurological deficits, including non-ambulatory tetraplegia or paraplegia and loss of deep pain perception, as well as in cases involving progressive deterioration. It may also be indicated when serial neurological monitoring is impractical or when the fastest feasible functional recovery is a priority. These indications reflect, in an HNPE-specific context, the broader patient-selection principles outlined in the ACVIM consensus statement on acute thoracolumbar intervertebral disc extrusion [25]. MRI findings, including the degree of compression and any intramedullary T2-weighted hyperintensity, should be considered alongside these clinical criteria, although no imaging threshold has yet been validated [5].

### 8.5. Cost Considerations

Cost is rarely reported explicitly in the HNPE literature, yet it often decides an owner’s choice in practice. Conservative management avoids the costs of anaesthesia, surgery and inpatient hospitalisation, and in appropriately selected cases achieves high recovery rates, which makes it a cheaper option that need not be regarded as a compromise [4,5], although equivalence between the two approaches has not been demonstrated (Section 8.3). Surgical decompression carries a higher upfront cost but resolves cord compression more quickly, and stays the only appropriate choice once deficits are severe or progressive. At that point, the cost of a delayed or failed conservative trial, in both clinical and financial terms, can outweigh whatever was saved by avoiding surgery in the first place.

## 9. Prognosis and Outcome

Taken as a whole, the prognosis for dogs with HNPE, whether treated surgically or conservatively, is good in most reported cases, with the majority regaining the ability to walk within days to a few weeks [4,5,10]. Recovery time appears to be associated mainly with the presence and length of intramedullary T2-weighted hyperintensity, rather than with the severity of the initial deficits or the degree of compression on imaging [5]. As with other acute myelopathies, dogs that have lost deep pain perception carry a more guarded prognosis, though this subgroup has not been studied in detail specifically within HNPE. In the closely related non-compressive group, deep-pain-positive paraplegic dogs with fibrocartilaginous embolic myelopathy or ANNPE regain ambulation far more often than deep-pain-negative dogs, and a machine-learning model built specifically for deep-pain-negative dogs undergoing decompressive surgery has begun identifying which of these more guarded cases still go on to recover [47,48]. Prolonged absence of nociception does not necessarily preclude a favourable outcome. A prospective study of dogs that had lacked nociceptive responses for more than 96 h before undergoing hemilaminectomy demonstrated that meaningful functional recovery remained achievable in a proportion of cases [49].

Comparing lesion types directly, a recent retrospective analysis found similar ambulation recovery rates between dogs with compressive disc extrusions and those with presumptive contusive, non-compressive lesions such as ANNPE, despite the two groups being managed very differently. That echoes a theme running through this review: outcome in these non-Hansen-type presentations seems to be driven more by the biology of the lesion than by the treatment chosen [50]. Structured rehabilitation appears to help dogs reach that outcome faster and more completely. Controlled and case-series data on intensive multimodal neurorehabilitation after severe disc extrusion surgery, on continued rehabilitation for dogs still neurologically incomplete months after surgery, and on physiotherapy with supportive standing devices to encourage spinal walking in deep-pain-negative paraplegic dogs all point to a role for structured aftercare alongside whichever primary treatment is chosen [51,52,53]. Long-term recurrence looks to be uncommon across the reported series, though follow-up periods vary considerably between studies, and recurrence itself has not been systematically assessed.

## 10. Conclusions and Future Directions

HNPE is now a reasonably well-characterised, and probably under-recognised, cause of acute spinal cord compression in dogs. It deserves a place in the differential diagnosis of peracute paresis or paralysis, particularly in small-breed dogs with cervical signs and little or no spinal pain. Recognising it on MRI and telling it apart from classic Hansen type I/II disease and from ANNPE has real consequences for how a case is managed. The comparative evidence shows that most dogs with compressive HNPE recover with either conservative management or surgical decompression, which makes medical treatment a reasonable and potentially more cost-effective option in carefully selected cases, particularly when surgery is not available or is declined. This is not the same as proven equivalence. The comparative studies are retrospective and non-randomised; treatment choice may have been influenced by neurological severity and MRI findings; the lack of significant differences partly reflects small numbers, and very few severely affected dogs have been studied. Prospective randomised trials are still lacking. Furthermore, thoracolumbar HNPE has received considerably less research attention than its cervical counterpart, while the distinction between HNPE and ANNPE remains inconsistently defined across published studies. These limitations highlight the need for larger, prospective investigations to establish more robust treatment recommendations and clarify the clinical boundaries between these conditions. Future work would do well to prioritise adequately powered prospective, ideally randomised, multicentre studies stratified by initial neurological severity and MRI findings, with standardised outcome measures and follow-up intervals, better characterisation of thoracolumbar cases, clearer criteria for choosing between conservative and surgical management informed by findings such as the 2025 exercise restriction data discussed above, genotyping of affected dogs for FGF4 retrogene insertions, and a better understanding of what actually triggers extrusion of non-degenerate nuclear material in the first place.

## Figures and Tables

**Table 1:** Comparison of cervical and thoracolumbar hydrated nucleus pulposus extrusion (HNPE) in dogs.

Feature	Cervical HNPE	Thoracolumbar HNPE
Frequency	Most reported cases (33 of 36 dogs in an early comparative series) [5]	Fewer reported cases overall, although thoracolumbar cases predominated in one referral series [5,6]
Typical signalment	Small and toy breeds, non-chondrodystrophic and chondrodystrophic; middle-aged to older, though juvenile cases occur [1,8,10]	Small breeds predominate; chondrodystrophic breeds more represented than in cervical HNPE, but less than in thoracolumbar Hansen type I extrusion; juvenile to adult dogs [6,7,18]
Pain at presentation	Often absent or mild; the majority of dogs are non-painful on examination [10]	Often mild; less hyperaesthesia than in thoracolumbar Hansen type I extrusion [7], but more often painful than cervical HNPE in one series [6]
Neurological signs	Peracute tetraparesis to tetraplegia; impaired voluntary urination in a minority of dogs [4,5]; respiratory compromise in some severely affected dogs [8,10]	Peracute paraparesis to paraplegia [6,7]; frequency of bladder dysfunction not compared with cervical HNPE
MRI appearance	Well-marginated, seagull-shaped extradural mass; disc of origin usually normal or near-normal [1,10]	Same well-marginated pattern described, though comparative imaging series are still limited [6,7]
Treatment response	High recovery rates with both approaches in the two early comparative cohorts, although equivalence has not been demonstrated [4,5]	No difference in outcome between conservative and surgical treatment in one series, although thoracolumbar cases were more often treated surgically [6]
Prognosis	Good in most reported cases; recovery time associated with intramedullary T2-weighted hyperintensity rather than with location or treatment [4,5,10]	Comparably good; all dogs ambulatory at follow-up in one series, with no difference in outcome between locations [6]

**Table 2:** Comparative diagnostic value of MRI and CT (with or without myelography) in HNPE.

Feature	MRI	CT/CT-Myelography
Soft-tissue contrast	Excellent; distinguishes extruded material, spinal cord and disc of origin directly [1,10]	Limited on plain CT; HNPE material is often close to isoattenuating with the spinal cord [30,31]
Detection of the seagull sign	Directly visible on T2-weighted sequences [1,10]	Not reliably seen without myelographic contrast [30,31]
Assessment of disc of origin	Readily assessed; usually normal or near-normal in HNPE [1,10]	Harder to characterise fully, even with myelo-CT, in subtler cases [31,32]
Need for contrast administration	Not required for lesion detection	Myelography or myelo-CT usually needed for confident diagnosis [30,31]
Availability and cost	Less widely available; higher cost; general anaesthesia required	More widely available in general practice; typically faster and less costly
Practical role	Preferred modality whenever accessible, especially with marked or worsening signs [1,10]	A reasonable fallback where MRI is unavailable or contraindicated, with the limitations above kept in mind [30,31,32]

**Table 3:** Comparative features of HNPE, classic Hansen type I and type II intervertebral disc disease, and ANNPE in dogs.

Feature	HNPE	Hansen Type I (Extrusion)	Hansen Type II (Protrusion)	ANNPE
Typical signalment	Small/toy and non-chondrodystrophic breeds; middle-aged to older dogs [1,8,10]	Chondrodystrophic breeds (e.g., Dachshund); young to middle-aged [2]	Non-chondrodystrophic, larger breeds; older dogs [2]	Large, active/athletic breeds; any age [2,3]
Onset	Peracute, often during normal activity	Acute, may follow exertion	Chronic, progressive	Peracute, typically during exercise/trauma
Spinal pain	Often absent or mild (~75% non-painful in cervical cases) [10]	Usually marked	Variable, often mild	Often transient, absent by presentation [2,3]
Disc degeneration	Minimal/absent at extrusion site [1,10]	Marked (chondroid metaplasia) [2]	Marked (fibroid metaplasia) [2]	Minimal/absent [2,3]
MRI appearance	Well-marginated, T2-hyperintense/T1-iso-hypointense extradural material (“seagull sign”); normal or near-normal disc of origin [1,10]	Heterogeneous, often T2-hypointense extradural material; degenerate disc [2]	Broad-based, T2-hypointense bulge; degenerate disc [2]	Intramedullary T2-hyperintensity, no/minimal extradural compression; “missing disc” may be seen [2,3]
Cord compression	Present, variable severity (0–62% reported) [5,10]	Present, often marked	Present, mild-moderate	Absent or minimal (non-compressive by definition) [2,3]
Treatment	Conservative management often as effective as surgery in appropriately selected cases [4,5]	Surgical decompression, especially with moderate-severe deficits [2]	Surgical decompression/fenestration; medical management for mild cases [2]	Supportive care and rehabilitation; surgery not indicated [2,3]

**Table 4:** Comparative summary of conservative management and surgical decompression for HNPE in dogs.

Aspect	Conservative Management	Surgical Decompression
Typical candidates	Ambulatory or only mildly non-ambulatory dogs, normo- to mildly hyperaesthetic, without progressive deterioration [4,5]	Severe, progressive or rapidly worsening deficits; owners wanting the fastest possible decompression [4,5]
What it involves	Strict activity restriction, analgesia as needed, nursing and bladder care, structured physiotherapy [25]	Ventral slot (cervical) or hemilaminectomy (thoracolumbar); removal of extruded material from the epidural space [10]
Reported neurological outcome	High recovery rates in the two comparative cohorts, with no significant difference from surgery; equivalence not demonstrated [4,5]	High recovery rates in the same cohorts [4,5]
Time to recovery	Similar to surgical treatment overall; associated mainly with intramedullary T2-weighted hyperintensity rather than treatment choice [5]	Similar to conservative treatment overall; associated mainly with intramedullary T2-weighted hyperintensity rather than treatment choice [5]
Main drawback	A minority of cases fail to respond and need delayed surgery; slower resolution of compression [4,9]	Anaesthetic and surgical risk; higher upfront cost; not necessary for most appropriately selected cases [42,43]
Practical takeaway	A reasonable first option in carefully selected, non-progressive cases [25]	Remains the appropriate choice once deficits are severe or progressive, or when conservative therapy fails [25]

## Data Availability

No new data were created or analyzed in this study. Data sharing is not applicable to this article.

## Abbreviations

The following abbreviations are used in this manuscript:

ACVIM	American College of Veterinary Internal Medicine
ANNPE	Acute non-compressive nucleus pulposus extrusion
CSF	Cerebrospinal fluid
CT	Computed tomography
FGF4	Fibroblast growth factor 4
HASTE	Half-Fourier acquisition single-shot turbo spin-echo
HNPE	Hydrated nucleus pulposus extrusion
IVDD	Intervertebral disc disease
MRI	Magnetic resonance imaging
